# Afterword: Science popularization, dictatorships, and democracies

**DOI:** 10.1177/00732753211073422

**Published:** 2022-05-18

**Authors:** Geert Somsen

**Affiliations:** Maastricht University, The Netherlands

**Keywords:** Science popularization, Francoist Spain, dictatorship, totalitarianism, science and ideology

## Abstract

This Afterword to the special section on Science Popularization in Francoist Spain draws general conclusions from its case studies. Most overarchingly, the different contributions show that popularization existed under this dictatorial regime, and hence does not require a Habermasian liberal-democratic public sphere. Four more specific lessons are also drawn, each shedding new light on either science popularization or dictatorial regimes. (1) Popularization has not only been a way to promote science, it has also been used to prop up dictatorial regimes by associating them with things scientific. (2) Totalitarian regimes are much less monolithic than they appear to be at the surface; they often harbor internal weaknesses and conflicts. (3) The study of science popularization in dictatorships can help open our eyes for comparable forms of propaganda in democracies. (4) Totalitarianism is best understood not as a universal phenomenon, but in its specific historical situatedness. Studying science popularization under Franco brings out the specific traits of this regime: the legacy of the Civil War, Spanish regionalism, and the international dependencies of the Francoist state.

## Introduction

This special section is about a subject that should not even exist. At least if we follow conventional thinking about dictatorships, “popular science” would be a near impossibility under such regimes. After all, popular science implies at least two prerequisites that are hard to imagine with dictatorial governments: (1) a deference to the truths of science, whether compatible with official ideology or not; and (2) a level of public participation, spontaneous and on its own terms, opening a space for free debate. Such a public sphere would, in Jürgen Habermas’ terms, seem to be a precondition for popular science, but is, in his theorizing of the subject, an occurrence specific to liberal democracy as produced by the Enlightenment. It should be a virtual absence in dictatorial states.^1^

The authors of this special section take on this observation and turn it into a couple of questions: To what extent *was* popular science possible under dictatorial regimes? And to what extent does such presence attest to the existence of a public sphere? In order to answer these questions, they focus on one particular regime, the rule of Generalissimo Francisco Franco over Spain for nearly half a century in what was a dictatorship with totalitarian ambitions.^2^ Furthermore, the authors introduce an important distinction to the analysis: that between popular science and science popularization. These two practices overlap but they are not identical. Popularization implies a popular*izer*: a person or institution that has privileged access to (bits of) science and communicates it (them) to the public. This presupposes a hierarchy and an essentially passive audience. The term popular science, by contrast, suggests a more active public, discussing, evaluating, perhaps even producing knowledge by or among itself. Today’s concept of citizen science would befit the latter category, but not the former.^3^

Exploring these issues in the context of the Francoist state, the authors end up questioning the Habermasian theory of the public sphere and the implied impossibility of popular science under dictatorship. By questioning this theory, they reveal hidden features of both science popularization and dictatorial regimes and produce new understandings of them. In this Afterword, I would like to discuss four such insights.

1. One general conclusion that we may draw from the three case studies collected here is that science popularization is not always about science. This is a finding that contradicts its own received wisdom. Within science and technology studies there is a long tradition of critical scrutiny of science popularization, which has shown, among other things, that the phenomenon entails much more than the dissemination of knowledge. Popularizing science is not just meant to inform the public, it also often entails soliciting their consent, asking citizens not merely to take note of, but to welcome, the findings of scientific research, and hence to value the institutions of science. As Brian Wynne has put it succinctly: all too often, “public understanding of science is automatically equated with public appreciation [. . .] of science.”^4^ True as this may be, the case studies presented here show something more. Within the science festivals, zoos, and popular magazines analyzed by Agustí Nieto-Galan, Miquel Carandell-Baruzzi, and Clara Florensa, popularization involved much more than selling science to receptive publics. It had political goals that often stretched beyond communicating scientific knowledge. Science was not the product in the transaction, but the vehicle – not the message, but the carrier of messages, and these messages invariably had something to do with the perception of the regime. Science is a suitable substrate for such communications since it comes with a variety of positive connotations. Not only is it readily associated with values like progress and modernity, civilization and sophistication, it also exudes objectivity, neutrality, and disinterestedness, and is therefore extremely persuasive for anything recommended by it. Hence the ubiquity of science in commercial advertisements, from dog food to day cream.

In the cases collected here, it was not commodities but institutions and ideologies that were being sold, and science proved an equally powerful referent. The 1955 Festival of Science analyzed by Agustí Nieto-Galan, for instance, helped Franco’s regime to bolster its image vis-à-vis the United States and Western Europe. In a series of exhibitions, lectures, and films, science was presented as an agent of material progress in modern technocratic societies (including Franco’s) and as a weapon against the pseudoscience harbored by “totalitarian” regimes – a term here reserved for communism. It was an agenda that helped to align Franco’s Spain with its new Western allies, who, until recently, had fought *against* authoritarian dictatorships and *with* the Soviet Union. At the festival, these alliances were reversed, and references to allegedly apolitical science helped facilitate that transition. An affiliated scientific congress further absorbed Spanish participants into a Western-dominated international atmosphere, solidifying the new national bonds through personal ties. Science, in other words, served as an ideological lubricant of particular constellations of international relations.

In the famous Barcelona Zoo, discussed in Miquel Carandell-Baruzzi’s contribution, popular science was an instrument for domestic propaganda. After thorough renovations following the latest insights on animal display, the zoo emerged in 1957 as a recognizably “modern” institution where public leisure was combined with scientific research. The modernity was part of a general campaign to revamp the city of Barcelona by its new mayor, José María de Porcioles. The Franco regime had installed him especially to put an end to the local resistance of student protests and tram strikes in previous years, and one way to placate popular opposition (besides brutal oppression) was public entertainment through “peaceful science.” But the zoo had more specific propagandistic aims as well. Visitors, especially children, were instructed how to behave on the premises in the belief that respect for animals would lead to respect for fellow human beings and obedience to the given social order. They were also asked to contemplate the wild variety of life forms as this would stimulate religious feelings and awe for God’s Creation: “the Zoo is a good catechism,” providing a proper Catholic education.^5^ All of this messaging was deliberately programmed to boost the regime’s image and appease its subjects.

Clara Florensa also shows how science popularization served to provide ideological backing for the Francoist state, yet her contribution goes further by revealing how it was mobilized *within* the regime by one faction against another. Under the dominant ideology of National Catholicism, both factions agreed that Darwinian evolution needed to be publicly rejected for its associations with materialism and hence communism (as well as, some claimed, Western consumerism). But they disagreed about strategy. According to the Falangists, the battle could be fought out in the open as a continuation of the Civil War, and Darwinian ideas and their proponents could be subjected and cleared of their dangerous elements. A group of reactionary “traditionalists,” however, claimed that the war had already been won, and that defeated ideas and their advocates should be hidden from public view so as to prevent “confusion” among the Spanish population. Their popular articles avoided any mention of Darwin or of Spanish Darwinists and presented a single Catholic biology. In the factions’ conflicts, the subject was evolutionary theory, yet the issue was how to deal with the legacies of Republican Spain.

2. This brings me to an aspect of totalitarianism revealed here that remains hidden in more traditional views of the phenomenon.^6^ They tend to present totalitarian regimes as more or less hermetic systems in total control over individuals and populations. But the case studies here chip away at the notion of totalitarian states as absolute and unified power blocks.^7^ Most clearly, Clara Florensa’s article reveals important fractures within the Franco regime that battled each other through their dealings with science. These culture wars, in effect, opened up a kind of public sphere where politics was not directly debated, but covertly through discourse on science. Miquel Carandell-Baruzzi shows that the successful launch of Barcelona’s new and modern zoo in fact attests to the regime’s lack of control of its population and its earlier inability to crush opposition. Science was here a way to discipline unruly citizens. Agustí Nieto-Galan, finally, demonstrates how weak the Francoist state was internationally after the Second World War, and how dependent on new foreign alliances. Both Spain and the Western allies needed to employ serious ideological gymnastics to justify their cooperation, and representations of science helped them to make those moves. In all these instances, the study of science popularization has helped to expose the cracks and the weak spots in allegedly uniform and total power.

3. But if totalitarian regimes are less total than they have been thought to be and less unified than they present themselves to be, then their characteristics may be less unique as well, and shared with other kinds of states. We may wonder if the uses of popular science found in this special section might also occur in contemporary democracies. Current research confirms that the answer is yes. Jaume Sastre has examined science popularization in the United States during the 1930s through 50s and encountered comparable strategizing.^8^ Especially in the years prior to the Second World War, concern was rising that the public started to associate science too much with communist societies. Fellow-travelers regularly sang the praises of the Soviet Union’s achievements in raising standards of living – successes that many of them attributed to its vast employment of scientific methods of production, management, and government. Capitalist societies, they claimed, lagged behind in their uses of science, and the Great Depression showed the complete irrationality of their modes of production. In 1938 and 1939, the Rockefeller Foundation gathered a number of science popularizers and government officials to see how they could redress the balance. The old association of science with shiny technologies and fancy gadgets was tainted by the crisis, and so plans were proposed to present science as an ideological ally to liberal values and democracy.

Similar moves were made a few years later in a different context – that of London shortly after the Blitzkrieg – as I have found in my own research.^9^ Here, actual campaigns were launched to present science as a “foundation” of democracy and the natural enemy of fascist Germany. Brochures were printed mocking the Nazis’ book-burnings, their racial biology, and their Aryan physics (which in fact had little Nazi support), while cartoons with such imagery were dropped from airplanes over occupied Europe. In the period before D-Day, propaganda makers began to worry that the people in Normandy and its environs might not see the British invaders as liberators but as another occupying power, so in order to spread a more positive image of Britain, they advertised British society (through pamphlets and radio shows) as modern and attractive and particularly science-minded. Here too science connoted democracy, freedom of speech and thought, and anti-irrationality. Just like in the United States and Spain, science was popularized as a vehicle for political messaging and ideological persuasion. Therefore, we can observe that what is conspicuous in the totalitarian regimes discussed in this special section may also be found in democratic societies.

4. Should the conclusion then be that there is nothing special about science popularization under totalitarianism? My hunch is that that would go too far – the differences with democracies were perhaps not absolute but they were not negligible either. What we need is a thorough historicization of totalitarianism, as Timothy Snyder has called for in his grand study *Bloodlands* (2010).^10^ Earlier analysts of the phenomenon, like Hannah Arendt, developed general theoretical models that took totalitarianism as a stage or condition that societies anywhere could assume. Although Arendt’s analysis started by following a particular historical pathway, its endpoint (the state of totalitarianism) transcended the specific places from which it grew.^11^ Snyder, by contrast, argues that we need to consider the confluences of particular historical developments if we are to understand the specificities of totalitarian regimes and their actions – in his case the separation, cooperation, and then conflict of the Soviet and Nazi states in their borderlands. If we apply such an approach to the Francoist state, the particularities of Spanish developments in the early to mid twentieth century become visible. Clara Florensa’s contribution, for example, clearly bears out how the prehistory of the Civil War and the legacy of Republican Spain left their traces in the shape that totalitarianism took. Similarly, Spain’s historic regionalism and uneven support for Franco become very evident in Miquel Carandell-Baruzzi’s analysis. And the regime’s postwar isolation and shifting of alliances are an important background in Agustí Nieto-Galan’s account. What the three case studies collected here show, perhaps most generally, is that totalitarianism deeply affected science popularization under Franco, but that it did so not by either enabling or blocking it, but by shaping its particular forms and contents in ways specific to interwar and postwar Spain.

## Footnotes

1 Jürgen Habermas, *The Structural Transformation of the Public Sphere: An Inquiry into a Category of Bourgeois Society* (Cambridge, MA: MIT Press, 1991). For a discussion of the relation of Habermas’ analyses to dictatorships, see the Introduction to this special section.

2 For a current discussion of the authoritarian/totalitarian nature of political regimes (especially in regard to Francoism), see Ismael Saz, Zira Box Varela, Toni Morant, and Julián Sanz (eds.), *Reactionary Nationalists, Fascists and Dictatorships in the Twentieth Century. Against Democracy* (London: Palgrave Macmillan, 2019).

3 For problematizations of the concepts of popular science and science popularization, see, besides the Introduction to this special section, Jonathan R. Topham, “Rethinking the History of Science Popularization/Popular Science,” in Faidra Papanelopoulou et al. (eds.), *Popularizing Science and Technology in the European Periphery, 1800–2000* (Aldershot: Ashgate, 2009), pp.1–20; Stephen Hilgartner, “The Dominant View of Popularization: Conceptual Problems, Political Uses,” *Social Studies of Science* 20(4) (1990): 519–39; Steven Shapin, “Science and the Public,” in R. C. Olby, G. N. Cantor, J. R. R. Christie, and M. J. S. Hodge (eds.), *Companion to the History of Modern Science* (London, New York: Routledge, 1990), pp.990–1007; and Agustí Nieto-Galan, “Antonio Gramsci Revisited: Historians of Science, Intellectuals, and the Struggle for Hegemony,” *History of Science* 49 (2011): 453–78.

4 Brian Wynne, “Public Understanding of Science,” in Sheila Jasanoff, Gerald E. Markle, James C. Petersen, and Trevor Pinch (eds.), *Handbook of Science and Technology Studies* revised edition (Thousand Oaks, London, New Delhi: SAGE Publications, 1995), pp.361–88: 362.

5 1957 zoo flyer, quoted in Miquel Carandell-Baruzzi, “Animals for the Mayor: Barcelona’s Zoo in the Making of Local Policies and National Narratives (1957–73),” *History of Science* 60 (2022): xx–xx, a. contribution in this special section.

6 See e.g. Carl Friedrich and Zbigniew Brzezinski, *Totalitarian Dictatorship and Autocracy* (Cambridge, MA: Harvard University Press, 1965) and Hannah Arendt, *The Origins of Totalitarianism* (New York: Schocken Books, 1976).

7 The case studies also undermine traditional scholarship’s absolute distinction between totalitarian and authoritarian states, as Clara Florensa and Agustí Nieto-Galan make clear in the Introduction to this special section.

8 Jaume Sastre-Juan, “Philanthropy, Mass Media and Cultural Hegemony: The Rockefeller Foundation and the Politics of Science Popularization in the 1930s,” in Massimiliano Badino and Pietro Daniel Omodeo (eds.), *Gramsci Today: Cultural Hegemony in a Scientific World* (Leiden: Brill, 2021), pp.297–318.

9 Geert Somsen, “Science and Democracy in British World War II Propaganda,” unpublished manuscript.

10 Timothy Snyder, *Bloodlands: Europe between Hitler and Stalin* (New York: Basic Books, 2010).

11 Arendt, *Origins of Totalitarianism* (note 6).

